# Cognitive Functioning Influences Mortality Risk Among Older Adults with COPD

**DOI:** 10.3390/healthcare12222220

**Published:** 2024-11-07

**Authors:** Srikanta Banerjee, Jagdish Khubchandani, Elizabeth England-Kennedy, Rhonda McIntyre, Karen Kopera-Frye, Kavita Batra

**Affiliations:** 1 College of Health Sciences, Walden University, Minneapolis, MN 55401, USA; srikanta.banerjee2@mail.waldenu.edu; 2 College of Health, Education, and Social Transformation, New Mexico State University, Las Cruces, NM 88003, USA; jagdish@nmsu.edu (J.K.); eengken@nmsu.edu (E.E.-K.); kfrye@nmsu.edu (K.K.-F.); 3 Department of Pediatrics and Office of Dean, Ross University School of Medicine, St. Michael 11093, Barbados; rmcintyre@rossu.edu; 4 Department of Medical Education, Kirk Kerkorian School of Medicine at UNLV, University of Nevada, Las Vegas, NV 89106, USA; 5 Office of Research, Kirk Kerkorian School of Medicine at UNLV, University of Nevada, Las Vegas, NV 89106, USA

**Keywords:** cognition, older adults, COPD, mortality, aging

## Abstract

Background/Objeectives: Chronic Obstructive Pulmonary Disease (COPD) is a leading cause of mortality in the United States (U.S.), with rates varying by disease severity, comorbidities, and sociodemographic factors. Cognitive impairment has been independently associated with increased mortality, but has not been well studied in relation to COPD despite being a frequently overlooked comorbidity in COPD patients. The purpose of this nationwide study was to assess the relationship between low cognitive performance and the risk of mortality among older adults with COPD while adjusting for major sociodemographic and health-related characteristics. Methods: This study utilized the 1999–2002 National Health and Nutrition Examination Survey (NHANES) and the respiratory mortality data of noninstitutionalized US adults aged over 65 years. Survival curves showing the combined effect of cognitive decline and COPD using the Kaplan–Meier product-limit method to estimate the percent survival of the subject at each point in time were used. Results: The final sample included 2013 older adults, with 39.1% showing low cognitive performance and 12.7% having COPD. Those with low cognitive performance were older, less educated, had lower income, were more likely to be racial/ethnic minorities, and had a history of cardiovascular diseases (CVD); they were also more likely to have COPD or chronic kidney disease (CKD). The adjusted hazard ratio for respiratory-related mortality risk was highest for individuals with both COPD and low cognitive performance (hazards ratio = 8.53), people with COPD alone also had a higher respiratory-related mortality risk (hazards ratio = 4.92), but low cognitive performance alone did not significantly increase respiratory-related mortality risk. Conclusions: These findings provide clearer insights into how cognitive impairment affects mortality risk in older adults with COPD and we discuss potential strategies to address this dual chronic health challenge effectively.

## 1. Introduction

Chronic Obstructive Pulmonary Diseases (COPDs) are among the leading causes of death in the United States (U.S.). While the prevalence of COPD declined in the U.S. from 1990 to 2011, it is estimated that in recent times (2012–2021), the rates may have remained stable with a projection that the U.S. has and will have one of the largest economic burdens from COPD [1,2,3]. Specifically, within the past decade, the rates and disparities for some groups have increased (e.g., older adults and those living in rural areas), and sociodemographic disparities have also been observed for mortality due to COPD [2,4,5]. Estimates since the COVID-19 pandemic are yet to be confirmed in the U.S. given the higher rates of COVID-19-related hospitalizations and deaths among individuals with COPD. However, another recent report estimates that more than 100,000 adults died of COPD in the past year, and more than 14 million are currently living with COPD [2,5,6,7].

The causes of mortality among COPD patients vary by disease severity, sociodemographic characteristics, medical history (e.g., comorbidities), health profile, and lifestyle behaviors [5,8,9,10]. In general, respiratory failure is considered the major cause of death among those with very severe or advanced COPD, while comorbidities such as cardiovascular disease and pulmonary cancers are the causes of death among individuals with mild to moderate COPD [9,10,11,12]. However, some major studies have shown findings deviating from these general trends. For example, a large cohort study of more than 300,000 adults from the U.K. with COPD found that among the 97,882 who died during follow-up, 25.7% died due to COPD-related illnesses, and 23.3% died due to cardiovascular diseases (CVDs). Interestingly, the investigators also found that COPD phenotype, severity, and frequency were significantly associated with all-cause mortality [11]. Another large cohort of more than 12,000 adults from Copenhagen found that among individuals with severe COPD at baseline, only a fourth had COPD listed as a cause of death upon follow-up, but comorbidities (e.g., CVD) were more likely to be listed as a cause of death [13]. Given these findings and the substantial underreporting of COPD as a cause of death, more precise estimates on causes and determinants of mortality among those with COPD are needed to prevent health loss and premature mortality [11,12,13,14].

With advancements in treatment and the chronic nature of the disease, most individuals with COPD in Western countries (e.g., the US) die after the age of 65 years. This trend further precludes the precise estimation of causes of death and contribution of risk factors to mortality among those with COPD as older adults have numerous other risk factors for mortality (e.g., comorbidities, polypharmacy, aging-related changes, etc.) [4,5,6,7,10,11,12]. Studies have continued to investigate risk factors for mortality among older adults with COPD. Age, male sex, body weight, smoking, dietary patterns, alcohol use, diabetes, physical inactivity, depression, anxiety, and comorbidities are some of the factors that have been frequently examined in relation to the major causes of death among those with COPD (i.e., respiratory, cardiovascular, or cancer deaths) [10,11,12,15,16,17,18,19]. Recently, there has been a lot of interest in the association between COPD and cognitive performance with some scholars suggesting that cognitive impairment could be among a common and frequently overlooked comorbidities in those with COPD [19,20,21,22,23]. The earliest meta-analysis on this topic included 14 studies (3 cohort, 11 cross-sectional) to report that COPD patients had a higher risk of cognitive dysfunction than controls (OR = 1.72; 95%CI = 1.12–2.65) and COPD exacerbations were strongly linked with cognitive decline [20]. This was followed with another meta-analysis by Yohannes and colleagues which included 23,116 individuals with COPD across 14 studies. The authors found that the pooled average age for individuals with COPD worldwide was 66.3 years, the pooled prevalence of mild cognitive impairment in COPD patients was 25% (95%CI = 23–42%) and any cognitive impairment was 32% (95%CI: 18–38%) [21]. More recently in 2022, two meta-analyses found a significant relationship between COPD and cognitive impairment [22,23]. The first one by Wang and colleagues included 428,030 participants (n = 6 cohorts) and found that compared to those without COPD, patients with COPD had a higher risk of cognitive impairment upon follow-up (RR = 1.30, 95%CI = 1.13–1.49) [22]. The second one by Zhao and colleagues included 10 studies with 71,174 COPD patients and 22,082 controls to report that there was a significant association between COPD and mild cognitive impairment incidence risk (OR = 2.11, 95%CI: 1.32–3.38) [23]. Despite these findings, the long-term impact of cognitive function on mortality risk among those with COPD has not been well explored. Therefore, the purpose of this nationwide study was to assess the impact of low cognitive performance on the risk of mortality among older adults with COPD after adjusting for major sociodemographic and health-related characteristics.

## 2. Methods

### 2.1. Study Participants and Procedures

We used the 1999–2002 National Health and Nutrition Examination Survey (NHANES) data collected by the National Center for Health Statistics (NCHS) [24,25]. This annual nationwide study was constructed to evaluate the health of adults in the United States using consolidated data from interviews and physical exams. Our sample is representative of noninstitutionalized US adults aged over 65. Vital status was determined by linking the NHANES 1999–2002 data with public-use mortality files from the National Death Index (from the date of survey participation through 31 December 2019) [24,25,26,27]. Respiratory mortality was ascertained by the NCHS through a probabilistic match between NHANES participants and National Death Index (NDI) death certificate records encompassing chronic lower respiratory diseases (J40–J47), influenza, and pneumonia (J10–J18). Data are available for public use through the CDC website, and details about this data have been extensively published [8,24,25,26,27,28]. The procedures and protocols for NHANES were approved by the NCHS before data collection. We received additional ethical approval through the Walden University IRB for data analysis using publicly available files.

### 2.2. Measures

For cognitive function, the Digit Symbol Substitution Test (DSST) was administered to older adults through the NHANES survey, 1999–2002 [24,25]. This test is a component of the Wechsler Adult Intelligence Test. The utility of the DSST as a clinical tool in neuropsychology was first shown in early 1900s due to this tests ability to reliability distinguish patients with brain damage from healthy controls during the screening of soldiers. The use of the DSST widened after becoming incorporated in the Wechsler–Bellevue Intelligence Scale (WBIS), developed in 1930s. The DSST is a highly valid tool that is sensitive to the presence of cognitive dysfunction and also, to change in cognitive function across a wide range of clinical populations [27,29]. In the current NHANES nationwide assessment, the DSST assessed response speed, sustained attention, visual-spatial skills, and associative learning and memory. The test consisted of a coding exercise where individuals print symbols that are matched with numbers, identified in a key, for 133 situations over 120 s. The score consists of the number of correct symbols printed during the allotted time frame (minimum: 0 and maximum: 133). This test has been used widely in epidemiological studies and extensive details on the administration of these tests and performance within NHANES can be found elsewhere [24,25,27,29]. We dichotomized the categories into the bottom 25th percentile classified as having low cognitive performance (vs. 75th percentile). 

COPD status was ascertained through the NHANES question asking, “has a doctor or other health professional ever told you that you have [disease]?”. Physician-diagnosed COPD was defined as a positive response to either chronic bronchitis or emphysema. Similarly, cardiovascular disease history was determined by the self-reported diagnosis of coronary heart disease, angina, stroke, congestive heart failure, or myocardial infarction. For chronic kidney disease (CKD), the glomerular filtration rate was derived from the Cockcroft-Gault equation using measured creatinine levels. 

Major health-related and demographic variables were also included in this analysis. Data on body mass index (BMI) was derived from measured height and weight and categorized into four groups (i.e., BMI < 25 kg/m^2^ = normal weight; BMI = 25–29 kg/m^2^ = overweight; BMI = 30–39.9 kg/m^2^ = obese; and participants with BMI > 40 kg/m^2^ were considered severely obese). For the multivariate models, obesity was dichotomized and considered present for BMI ≥ 30 kg/m^2^ and considered absent for the rest. A two-variable indicator of current smoking status was created with “nonsmoker” (coded 0) and “smoker” (coded 1). The subject was considered a “smoker” if s/he reported “yes” to the question, “Have you smoked at least 100 cigarettes in your entire life?” and answered “every day” and “some days” to the question, “Do you now smoke cigarettes...”. 

For demographic variables to be considered as covariates, age, sex, education, income, and ethnicity were utilized. Ethnicity was divided into “Non-Hispanic White”, “Non-Hispanic Black”, “Hispanic”, “Asian”, and others. Education-level data was categorized into three groups: “less than High School” versus “High School graduate” versus “Some College or above”. Income information was computed by the poverty income ratio (PIR), which is also an indicator of income relative to the economic needs of a household. This is a polytomous variable that was achieved by calculating annual fluctuations in household size and cost of living and monitoring the consumer price index concerning household income and federally established poverty limitations. PIR levels were defined as low income (PIR < 1), middle income (1 ≤ PIR < 4), and high income (PIR ≥ 4) and dichotomized for analysis with a cutoff point of 1.

### 2.3. Statistical Analysis

We weighted demographic variables to approximate distributions in the US by using the provided sample weights (to account for oversampling of certain groups, unequal probabilities of selection, and non-response). Normal distributions of values were assessed by using the Shapiro–Wilk test. Categorical variables were expressed as percentage values and analyzed using chi-square tests. Complex samples multiple Cox regression models were used to examine the risk of respiratory-related mortality based on COPD or cognitive decline after adjusting for covariates. Statistical analyses were conducted using the SAS System for Windows (release 9.3; SAS Institute Inc., Cary, NC, USA) and SUDAAN (release 9.0; Research Triangle Institute, Research Triangle Park, NC, USA). Additionally, we generated survival curves showing the combined effect of cognitive decline and COPD using the Kaplan–Meier product-limit method to estimate the percent survival of the subject at each point in time. Statistical significance for tests was considered at a *p*-level of <0.05.

## 3. Results

A total of 2013 older adults were included in the final sample where more than a third had low cognitive performance (39.1%) and more than a tenth (12.7%) had COPD. The average duration of follow-up for all study participants was 10.7 years. Table 1 provides data on the distribution of demographic characteristics of the study participants stratified by cognitive performance using bivariate analysis. Individuals with lower cognitive performance were statistically significantly more likely to be older, with lesser education and income, racial/ethnic minorities, and a history of CVDs. Also, individuals with lower cognitive performance were significantly more likely to have COPD or CKD. 

**Figure 1:**
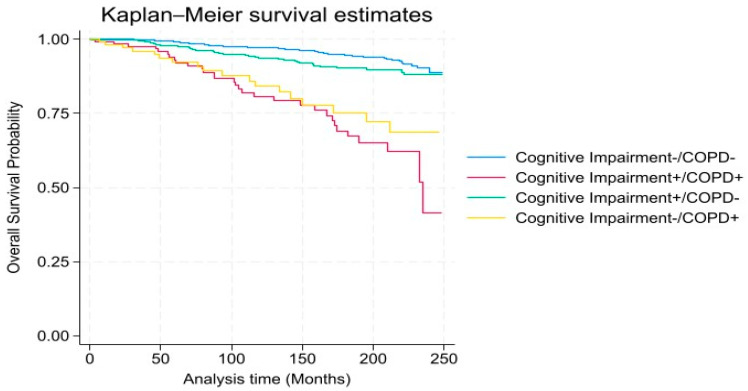
Respiratory-related mortality among individuals with and without cognitive impairment/COPD (x-axis: time in months, y-axis: overall survival probability).

Compared to those without COPD, the unadjusted hazard ratio (HR) for respiratory-related mortality risk among those with COPD was 4.93 (95%CI = 2.72–8.93, *p* < 0.001). However, compared to individuals without COPD, the adjusted HRs for individuals with COPD was 5.12 (95%CI = 2.84–9.24) after adjusting for demographic (age, gender, ethnicity, and PIR) and health risk factors (CKD, CVD, and obesity) [Table 2]. When stratified by groups based on COPD or cognitive performance, the adjusted HR was highest among individuals with both COPD and low cognitive performance [HR 8.53 (95%CI, 2.45–29.42)]. Also, individuals with COPD but without low cognitive performance had a significantly higher risk of respiratory-related mortality [HR 4.92 (95%CI = 1.92–12.57)]. Individuals with low cognitive performance alone did not have a significantly higher risk of respiratory-related mortality. As shown in Figure 1, there was a higher probability of respiratory-related mortality over time (mean = 10.7 years) among individuals with cognitive decline.

## 4. Discussion

In our analytic sample, we found that more than a tenth of older Americans have COPD and more than a third have some cognitive impairment, closely resembling the published estimates from other population-based U.S. studies [2,7,29]. Furthermore, our findings on the prevalence of COPD based on cognitive performance are in line with existing evidence on the association between cognitive impairment and COPD (15.3% of individuals with low cognitive performance had COPD vs. 11.7 of those who had normal cognitive performance) [20,21,22]. Above all, compared to their counterparts, the risk of respiratory-related mortality among individuals with COPD alone was nearly five times higher and among those with both COPD and low cognitive performance, the risk of respiratory-related mortality increased by nearly nine times. While the causal links need to be established through additional studies, certain hypotheses may explain these connections [11,12,18,19]. First, individuals with cognitive impairments may have problems with medication adherence, activity coherence, missed medical appointments and guidance, and poor compliance with treatments leading to premature mortality. Second, individuals with cognitive impairments may not be able to avoid risk factors for worsening COPD that could lead to poor health outcomes and mortality. Third, individuals with cognitive impairments (e.g., memory, cognitive flexibility, and visual processing) may not be able to self-manage their disease, remain isolated, or have profound disability and lower engagement in healthy behaviors, these factors could lead to worsening of both cognitive impairment and COPD increasing the risk of mortality. Fourth, cognitive impairments are independently linked with frailty, comorbidity progression and worsening, and higher risk of hospitalization; all of these factors could increase the risk of COPD-related mortality. Finally, the complex and reciprocal relationships between COPD and cognitive function must also be acknowledged. For example, COPD often results in reduced oxygen levels in the blood, which can affect cognitive function due to reduced oxygen supply to the brain. Also, COPD is associated with systemic inflammation, which can impact cognitive health as the systemic circulating inflammatory biomarkers may accelerate neurodegenerative processes. [9,11,12,18,19]. Beyond these mechanisms, and above all, the biological mechanisms in the relationship between COPD and cognitive impairment may have shared pathways leading to worsening health and higher risk of mortality (e.g., widespread inflammation, vascular and immune dysfunction, disruption of normal physiological processes, higher risk of other comorbidities, etc.). For example, it is fairly well established that individuals with COPD often have other major chronic health issues (e.g., cardiovascular disease, depression) that can further contribute to cognitive decline and increase the risk of mortality [8,9,21,28,30].

Before this study, it was suggested that cognitive dysfunction could be linked with increased mortality in acute hospital admissions even in those without COPD [19,30]. In fact, one small prospective study (n = 244 elderly COPD patients for 6-month follow-up) found no association between mortality and cognition (assessed by Mini-Mental State Examination) [31]. In contrast, another small prospective study (n = 149 COPD patients with 29 deaths after 32 months) found drawing impairment was associated with a significantly higher risk of mortality among severe COPD patients [32]. It should be noted that these studies had limitations that we tried to overcome in our analysis. First, these studies were from small, convenience, and healthcare facility-based samples including individuals with severe COPD. Second, these studies included a limited number of major health and sociodemographic variables limiting the reliability and validity of findings. Furthermore, the assessment for cognitive function was not as comprehensive in these studies compared to ours [31,32].

### 4.1. Implications for Research and Practice 

The results of our analysis also suggest major areas for continued research, especially with major recent developments and given the complex interplay between COPD, cognitive functioning, and mortality risk [6,33,34,35,36,37,38]. Firstly, due to the link of COPD with COVID-19 infections that has been well established for morbidity and mortality outcomes. Second, due to the ongoing research emphasis on post-COVID-19 outcomes (e.g., Long COVID, which has symptoms of lung disorders and cognitive impairments). Third, recent epidemiological evidence has linked modern-day lifestyles and exposures to both cognitive function and COPD and possibly shared mechanisms for the worsening of both (e.g., higher life expectancy, air pollution, altered diets, toxic chemicals in water, etc.). Fourth, recent data indicate that medication adherence rates among those with COPD may have declined post-COVID [33,34,35,36,37,38]. Well-designed mixed-methods longitudinal studies are needed to understand the complex relationship between COPD, cognitive functioning, and risk and protective factors related to mortality to ascertain temporal relationships, the effect of medication and other therapeutic interventions, the influence of sociodemographic variables, and relative contributions of various risk factors (e.g., lifestyle, comorbidities, and environmental dynamics). Such studies should include periodic and comprehensive measurements for both COPD and cognitive performance to make meaningful contributions to the existing literature. There are brief measures for both COPD and cognitive decline, and studies are needed to understand how these can be deployed in primary care settings, specialist care facilities, or residential/nursing homes attending to older adults for comprehensive and periodic screening of individuals with either disorder for better management [39,40]. Regarding compliance and medication adherence, studies should investigate risk factors both internal to individuals (e.g., medication beliefs and level of health literacy) and externally located (e.g., cost, access, and under- or misdiagnosis of COPD or suboptimal medication regimens) [41]. 

Critical to advancing our understanding of the relationship between cognitive performance, COPD, and increased mortality is the nature of healthcare practice and variation in approaches to patients among professionals [19,20,22,23]. For example, clinicians often may not know how well their patients understand treatment regimes. Existing studies have shown the impact of cognitive impairment on older adults as a barrier to medication adherence, a factor frequently ignored by providers. A related construct is the treatment burden or the effort a patient has to put in to manage their chronic conditions (e.g., physical and emotional ability, cost and access, knowledge and health literacy, etc.) [19,23,30,31]. Furthermore, cognitive function also affects the use of inhaled drugs, which translates into disease compensation and the severity of COPD symptoms also has a huge impact on treatment compliance [38,39,42]. Most critically, general practitioners may engage in polypharmacy or prescribe medications without a thorough understanding of cognitive function or COPD status (e.g., without spirometry); this could further worsen health outcomes and increase mortality risk [40,41,42,43,44]. Clinicians should be sensitized to the unique needs of elderly with COPD such as limited resources, lower health literacy, inability to easily gain or understand medical information, and phenotypic variations in COPD and cognitive decline. Addressing both respiratory and cognitive health in COPD management is essential for improving health outcomes, quality of life, and survival among individuals affected with these chronic health issues. 

There is a need for education of providers and social support individuals, including workshops and other educational formats that provide continuing education requirements for medical and health alliance personnel. Physician and other medical personnel training that addresses provider attitudes towards patients, beliefs about patients and these comorbid conditions, and knowledge about diagnosis and individualization of treatment regimens are needed for COPD and cognitive impairment. Caregivers can be educated using in-person workshops, telehealth, and phone-based apps on treatment approaches and communication techniques that they can use when providing support. Home Health Care Aides and Certified Nursing Assistants, social workers, and Community Health Workers can also aid in providing community education for healthcare management. These groups are especially valuable in providing outreach and support in rural and frontier areas. Employees of residential treatment settings are likely to need training on how best to monitor and address symptoms and signs of individuals with these specific co-morbidities. To summarize, the clinical focus for management of COPD among older individuals with lower cognitive functions (i.e., such as those include in this study) should be in certain major areas such as: environmental and lifestyle modifications, regular assessments and monitoring for COPD and cognitive functioning, pulmonary rehabilitation and cognitive support, medication provision and management to simplify regimes and maximize benefits, and finally, caregiver support and advanced care planning [19,20,21,22,23,42,43,44,45].

### 4.2. Limitations

The results of this study should be interpreted while keeping in view certain major limitations. First, except for mortality data, most of the data in this study were based on self-reported information, thereby posing a threat to reliability. Second, COPD positive status in this study was based on self-reports of participants in this nationwide data collection initiative of the U.S. CDC. This is a major threat to validity of our findings as there is no verification of the clinical diagnosis of COPD (i.e., based on spirometry results, especially the FEV1/FVC ratio or classification of disease severity using available validated tools or the GOLD classification). Also, potentially relevant information on COPD (e.g., treatment, lung function indices, disease duration, prescription medications used, etc.) was not available in the NHANES data. Such information could have influenced our analysis and results. Third, we only examined the risk of respiratory-related mortality among various groups based on COPD and cognitive functioning with a narrow population age group. Our analysis may give a limited view of the risk of overall mortality (e.g., from CVDs or other causes) among those with COPD and poor cognition. Additional prospective studies are needed to fully understand the impact of cognitive functioning on mortality risk among those with COPD along with examination of various sociodemographic groups. Fourth, while NHANES data are considered highly representative of the U.S. population, the generalization of our findings to other populations should be approached with caution (e.g., we did not have an adequate number of individuals from some specific racial/ethnic minority populations, most participants were female, and more than two-thirds had a high school diploma). Finally, the results suffer from all traditional limitations of cross-section study designs (e.g., inability to establish cause and effect relationships). 

## 5. Conclusions

This nationwide study has shown that there is an increased risk of respiratory-related mortality in older adults with COPD associated with low cognitive performance, as compared to those with COPD without cognitive impairment. This association may be attributed to factors that influence and/or are influenced by cognitive impairment and related to pathophysiologic processes as well as socio-demographic factors. In addition, there was a higher prevalence of low cognitive performance in patients with COPD as compared to those without COPD. This highlights the need for screening evaluations to ensure the early detection of low cognitive performance in older patients with COPD. This will help inform the initiation of targeted early interventions and help mitigate the risk of increased respiratory-related mortality.

## Figures and Tables

**Table 1:** Characteristics of study participants stratified by cognitive performance.

Characteristics	Total Population (*n* = 2013)	Low Cognitive Performance (*n* = 788)	Normal Cognitive Performance (*n* = 1225)
Age (SE) **	74.1 (0.21)	76.3 (0.32)	72.8 (0.26)
**Gender Female** (%)	58.1 (56.1–60.0)	55.2 (51.1–59.3)	59.2 (56.5–61.9)
Education Level (%) **			
Some High School	28.8 (24.6–33.1)	49.7 (42.9–56.4)	20.6 (17.1–24.0)
High School Grad	29.1 (25.5–32.6)	25.2 (20.0–30.4)	30.6 (27.0–34.2)
Some College or Above	42.1(37.8–46.4)	25.1 (19.2–31.0)	48.8 (44.2–53.5)
Family Poverty Income Ratio * (%) **			
Low Income < 1	13.5 (10.6–16.4)	28.5 (23.1–33.9)	7.5 (5.6–9.9)
Middle Income 1–4	62.8 (59.3–66.2)	61.6 (57.1–66.1)	63.2 (59.0–67.5)
High Income > 4	23.8 (20.0–27.6)	9.9 (6.9–14.1)	29.3 (24.9–33.8)
**Race/Ethnicity** (%)			
Non-Hispanic White	85.5 (81.4–89.7)	72.6 (64.2–81.0)	90.7(87.2–93.3)
Non-Hispanic Black	5.8 (4.1–8.1)	12.1 (7.2–17.0)	3.2 (2.3–4.5)
Hispanic	6.6 (3.4–12.4)	12.9 (4.4–21.5)	4.1 (2.0–8.1)
Other	2.1 (1.3–3.5)	2.4 (1.1–5.0)	2.0 (1.1–3.5)
**Diabetes** (%)	14.6 (13.0–16.2)	17.1 (11.8–22.4)	17.5 (12.1–22.9)
**Smoking Status** (%)	9.6 (8.1–11.3)	9.3 (7.0–12.2)	9.7 (7.8–12.0)
Cardiovascular Disease **	25.9 (22.9–28.8)	34.1 (28.9–39.4)	22.6 (19.6–25.7)
CKD Stages (%) **			
Normal to Mild ≥ 60	68.8 (66.3–71.2)	58.7 (52.3–65.1)	72.6 (70.1–75.1)
Moderate = 30–59	29.2 (26.9–31.60	37.3 (31.3–43.3)	26.1 (23.5–28.8)
Severe = 15–29	1.7 (1.1–2.6)	3.5 (2.2–5.4)	1.0 (0.5–2.3)
End Stage < 15	0.3 (0.1–0.7)	0.5 (0.2–1.6)	0.2 (0.1–0.8)
**Obesity Status** (%)			
Normal Weight BMI < 25	31.8 (29.1–34.5)	33.4 (27.5–39.2)	31.2 (27.7–34.8)
Overweight BMI = 25–29.9	39.2 (36.1–42.3)	37.1 (30.7–43.5)	40.0 (36.3–43.6)
Obese BMI = 30–39.9	26.1 (23.2–28.7)	27.2 (22.8–31.7)	25.5 (22.5–28.5)
Morbidly Obese BMI ≥ 40	3.1 (2.2–4.3)	2.3 (1.2–4.6)	3.3 (2.3–4.9)
COPD (%) *	12.7 (10.1–15.3)	15.3 (11.2–19.5)	11.7 (9.1–14.3)
**Respiratory-Related Deaths** (%)	10.2 (7.8–12.5)	10.2 (5.9–14.4)	10.2 (8.0–12.4)

Note. * *p* < 0.05 ** *p* < 0.01. Numbers with 95 CI indicate 95% confidence intervals for proportions.

**Table 2:** Risk of respiratory-related mortality among individuals aged 65 or older with COPD/low cognitive performance.

	Total Population HR (95%Ci) COPD vs. No-COPD	Low Cog+ COPD- HR (95%Ci)	Low Cog-COPD+ HR (95%Ci)	Low Cog+ COPD+ HR (95%Ci)
Comparison Groups	5.12 (2.84–9.24) **	0.97 (0.46–2.06)	4.92 (1.92–12.57) *	8.53 (2.47–29.42) *
Obesity	1.53 (0.93–2.53)	2.02 (1.23–3.31) *	2.29 (0.98–5.33)	0.65 (0.21–2.07)
Diabetes	1.29 (0.63–2.64)	1.51 (0.63–3.65)	0.88 (0.27–2.92)	1.21 (0.48–3.03)
Cardiovascular Disease	2.05 (1.18–3.56) *	1.35 (0.82–2.23)	2.68 (0.98–7.34)	1.26 (0.54–2.92)
Chronic Kidney Disease	0.87 (0.46–1.64)	0.70 (0.40–1.23)	1.14 (0.44–2.94)	0.77 (0.41–1.44)
Smoking	2.18 (1.01–4.71) *	1.90 (0.95–3.80)	2.60 (1.07–6.32) *	1.16 (0.29–4.60)
Age	1.07 (1.02–1.11) *	1.11 (1.04–1.18) *	1.04 (0.97–1.11)	1.07 (0.98–1.17)
Gender (Ref: Male)	0.77 (0.57–1.04)	0.54 (0.30–0.99) *	0.72 (0.47–1.09)	1.17 (0.44–3.12)
Education Level				
College and Beyond	Ref.	Ref.	Ref.	Ref.
High School Graduate	1.02 (0.54–1.92)	0.79 (0.30–2.11)	1.04 (0.42–2.56)	2.01 (0.30–13.61)
High School	2.38 (1.29–4.37) *	2.97 (1.38–6.40) *	2.43 (0.95–6.22)	2.47 (0.83–7.29)
**Ethnicity**				
Non-Hispanic White	Ref.	Ref.	Ref.	Ref.
Non-Hispanic Black	0.52 (0.20–1.36)	0.56 (0.18–1.75)	1.11 (0.16–7.52)	0.26 (0.05–1.46)
Hispanic	0.57 (0.25–1.31)	0.83 (0.30–2.31)	0.98 (0.16–6.22)	0.43 (0.19–0.99)
Other	1.91 (0.64–5.65)	1.82 (0.41–8.03)	--	4.73 (1.03–21.58) *
Family Poverty–Income Ratio (Ref: PIR ≥ 1)	1.00 (0.51–1.95)	0.57 (0.29–1.13)	0.62 (0.34–1.11)	1.16 (0.50–2.69)

Note. * *p* < 0.05 ** *p* < 0.01. HR (95Ci) indicates hazard ratios with 95% confidence intervals for the outcome (i.e., respiratory-related mortality). Ref indicates the reference group among each variable for comparison with other groups.

## Data Availability

Data files are publicly available through the National Center for Health Statistics (NCHS).
